# Introducing a new approach for modeling a given time series based on attributing any random variation to a jump event: jump-jump modeling

**DOI:** 10.1038/s41598-024-51863-5

**Published:** 2024-01-12

**Authors:** Ali Asghar Movahed, Houshyar Noshad

**Affiliations:** Department of Physics and Energy Engineering, Amirkabir University of Technology (Tehran Polytechnic), Hafez Avenue, P.O. Box 15875-4413, Tehran, Iran

**Keywords:** Mathematics and computing, Statistics

## Abstract

When analyzing the data sampled at discrete times, one encounters successive discontinuities in the trajectory of the sampled time series, even if the underlying path is continuous. On the other hand, the distinction between discontinuities caused by finite sampling of continuous stochastic process and real discontinuities in the sample path is one of the main problems. Clues like these led us to the question: Is it possible to provide a model that treats any random variation in the data set as a jump event, regardless of whether the given time series is classified as diffusion or jump-diffusion processes? To address this question, we wrote a new stochastic dynamical equation, which includes a drift term and a combination of Poisson jump processes with different distributed sizes. In this article, we first introduce this equation in its simplest form including a drift term and a jump process, and show that such a jump-drift equation is able to describe the discrete time evolution of a diffusion process. Afterwards, we extend the modeling by considering more jump processes in the equation, which can be used to model complex systems with various distributed amplitudes. At each step, we also show that all the unknown functions and parameters required for modeling can be obtained non-parametrically from the measured time series.

## Introduction

The analysis of stochastic processes has been a fundamental problem of interest in many research fields for decades. Examples of such processes include fluctuations of wind and solar power systems^1^, fluctuations in the porosity and permeability of porous media^2^, price fluctuations^3^ and heart rate fluctuations^4^, which range from physics to engineering, economics and medicine. The analysis and modeling of such stochastic processes is not only aimed at better understanding their physics, but also helps to predict their future state in a probabilistic sense.

In order to model complex systems non-parametrically, it is necessary to determine the properties and strength of the fluctuating forces from measured time series. This leads to the question: Given a fluctuating set of experimentally measured data, how should one uncover the features of the fluctuations, and construct a dynamical stochastic equation that can describe the random variation of the measured data set? So far, considerable progress has been made to answer this question. One of the first steps was taken by presenting the Langevin equation to describe the random evolution of continuous diffusive processes^5^. However, the boundaries of stochastic processes extend beyond purely diffusive processes, and often include other processes with jump discontinuities^6^. In recent years, with the aim of going beyond the limited scope of continuous processes, Langevin modeling has been improved. In this regard, by focusing on processes involving jump discontinuities, the classical Langevin equation was extended, and discontinuous processes were modeled via the jump-diffusion equation^7,8^. A jump-diffusion equation includes both diffusion contributions as well as random jumps, and is able to describe the random evolution of discontinuous processes^9^. On the other hand, when dealing with data sampled at discrete times, one encounters successive discontinuities along the trajectory of the sampled time series, even when the underlying path is continuous^10^. Therefore, in such a case, one cannot initially be sure which equation to use for modeling, unless the diagnostic criteria presented in this context are used, which also has its own difficulties^11^.

The aim of this article is to introduce and discuss a new dynamical stochastic equation in which any variation in the path of the sampled time series is attributed to a jump event, regardless of whether the given time series belongs to the class of continuous processes or not. We will discuss our dynamical stochastic equation in the next sections, but before that, let us first explain how continuous and discontinuous stochastic processes are defined mathematically.

By definition, a process $x(t)$ is called continuous if its Kramers-Moyal (KM) conditional moments ${K}^{\left(n\right)}\left(x,t\right)= \left\langle{{\left[x\left(t+dt\right)-x\left({\text{t}}\right)\right]}^{n}}\right\rangle{|}_{x\left(t\right)=x}=\int d{x}{\prime}{\left({x}{\prime}-x\right)}^{n} p({x}{\prime},t+dt|x,t)$, where $\left\langle{\dots }\right\rangle{|}_{x\left(t\right)=x}$ denotes averaging over the conditional distribution, satisfy in the following relations for small time increments $dt$^12^:

1 $$ \begin{gathered} K^{\left( 1 \right)} \left( {x,t} \right) = M^{\left( 1 \right)} \left( {x,t} \right)dt + {\mathcal{O}}\left( {dt} \right) \hfill \\ K^{\left( 2 \right)} \left( {x,t} \right) = M^{\left( 2 \right)} \left( {x,t} \right)dt + {\mathcal{O}}\left( {dt} \right) \hfill \\ K^{\left( n \right)} \left( {x,t} \right) = {\mathcal{O}}\left( {dt} \right) {\text{for}} n \ge 3, \hfill \\ \end{gathered} $$

where $\mathcal{O}\left(dt\right)$ contains all terms above the first-order of $dt$, which means that $\mathcal{O}(dt)/dt$ vanishes when $dt\to 0$. Also ${M}^{\left(n\right)}\left(x,t\right)$ are known as Kramers-Moyal (KM) coefficients, and defined by the following relation:

2 $${D}^{(n)}\left(x,t\right)={M}^{(n)}\left(x,t\right)= \underset{dt\to 0}{{\text{lim}}}\frac{1}{dt}{K}^{\left(n\right)}\left(x,t\right),$$

that can be estimated directly from measured data set^13,14^. It should be noted that these definitions of KM coefficients differ from common definitions in books or articles by a factor of $\frac{1}{n!}$^12,15^.

It is evident from (1) that for a continuous process only two KM coefficients ${M}^{\left(1\right)}\left(x, t\right)$ and ${M}^{\left(2\right)}\left(x, t\right)$ are non-vanishing when $dt\to 0$. As a result, with vanishing higher-order KM coefficients, especially ${M}^{\left(4\right)}\left(x,t\right)$, one can ensure that $x(t)$ is continuous in the statistical sense. (indeed according to the Pawula theorem when ${M}^{(4)}\left(x,t\right)$ vanishes, all other KM coefficients ${M}^{\left(n\right)}(x, t)$ for $n\ge 3$ will also vanish^12,16^).

### The Langevin equation

The Langevin Equation is a widely used equation for modeling a continuous diffusion process. The Langevin dynamics produces a continuous sample path, and has the following expression using the Itô’s calculus for stochastic integrals^17,18^:

3 $$dx\left(t\right)={D}^{\left(1\right)}\left(x,t\right)dt+\sqrt{{D}^{\left(2\right)}\left(x,t\right)}dW\left(t\right).$$

In this equation, $x(t)$ is the state variable of the process, and $\{W\left(t\right), t\ge 0\}$ is a scalar Wiener process. Additionally, ${D}^{\left(1\right)}\left(x,t\right)$ and ${D}^{\left(2\right)}\left(x,t\right)$ denote the first and second-order KM coefficients that are known as the drift and diffusion terms, respectively, and are obtained from Eq. (2) as follows:

4 $$ \begin{gathered} D^{\left( 1 \right)} \left( {x,t} \right) = M^{\left( 1 \right)} \left( {x,t} \right) = \mathop {{\text{lim}}}\limits_{dt \to 0} \frac{1}{dt} \left\langle{ \left[ {x\left( {t + dt} \right) - x\left( {\text{t}} \right)} \right]^{1} }\right\rangle |_{x\left( t \right) = x} \hfill \\ D^{\left( 2 \right)} \left( {x,t} \right) = M^{\left( 2 \right)} \left( {x,t} \right) = \mathop {{\text{lim}}}\limits_{dt \to 0} \frac{1}{dt} \left\langle{ \left[ {x\left( {t + dt} \right) - x\left( {\text{t}} \right)} \right]^{2} }\right\rangle |_{x\left( t \right) = x} \hfill \\ \end{gathered} $$

These coefficients are estimated directly from measured time series.

### The jump-diffusion equation

Many stochastic processes are not classified as continuous processes^19–22^, and therefore the use of the Langevin equation is not justified for them. In general, non-vanishing ${M}^{\left(4\right)}\left(x,t\right)$ means that there are discontinuities in the trajectory of the time series, and jump events have a very important role in the underlying process. It is therefore necessary to improve Langevin equation to model discontinuous processes. One of the best generalizations of the classical Langevin equation that can create a discontinuous sample path is written as follows^8,10^:

5 $$dx\left(t\right)={D}_{j}^{(1)}\left(x,t\right)dt+\sqrt{{D}_{j}^{(2)}\left(x,t\right)}dW\left(t\right)+\xi dJ\left(t\right),$$

where again $\{W\left(t\right), t\ge 0\}$ is a scalar Wiener process, while ${D}_{j}^{(1)}\left(x\right)$ and ${D}_{j}^{(2)}\left(x\right)$ are the deterministic drift, and the diffusion coefficients (the j index denotes jumpy behavior, and is used to distinguish these KM coefficients from those defined in continuous processes), and $J(t)$ is a Poisson jump process^23^. The jump has rate $\lambda (x,{\text{t}})$ and size $\xi$, which can have any symmetric distribution with finite even-order statistical moments, e.g. Gaussian distribution. It is shown that all the coefficients and parameters required in this modeling can be found directly from the measured time series by estimating the KM coefficients as follows^8,10^:

6 $$ \begin{gathered} M^{\left( 1 \right)} \left( {x,t} \right) = D_{j}^{\left( 1 \right)} \left( {x,t} \right) \hfill \\ M^{\left( 2 \right)} \left( {x,t} \right) = D_{j}^{\left( 2 \right)} \left( {x,t} \right) + \lambda \left( {x,t} \right)\left\langle {\xi^{2} } \right\rangle \hfill \\ M^{\left( n \right)} \left( {x,t} \right) = \lambda \left( {x,t} \right)\left\langle {\xi^{n} } \right\rangle {\text{for}} n > 2 \hfill \\ \end{gathered} $$

Assuming that $\xi$ is a random variable with a Gaussian distribution, i.e.$\xi \sim N(0, {\sigma }_{\xi }^{2})$ as well as using the relation $\left\langle{{\xi }^{2l}}\right\rangle =\frac{2l!}{{2}^{l}l!}\left\langle{{\xi }^{2}}\right\rangle^{l}$ for the Gaussian random variables in the last relation in Eq. (6) for $n=4$ and $n=6$, the amplitude of the jump ${\sigma }_{\xi }^{2}(x,t)$, and the rate of the jump $\lambda (x,t)$ are estimated to be as follows:

7 $$ \begin{gathered} \sigma_{\xi }^{2} \left( {x,t} \right) = \frac{{M^{\left( 6 \right)} \left( {x,t} \right) { }}}{{5M^{\left( 4 \right)} \left( {x,t} \right) { }}} \hfill \\ \lambda \left( {x,t} \right) = \frac{{M^{\left( 4 \right)} \left( {x,t} \right)}}{{3\sigma_{\xi }^{4} \left( {x,t} \right)}} \hfill \\ \end{gathered} $$

By obtaining the jump parameters ${\sigma }_{\xi }^{2}(x,t)$ and $\lambda \left(x,t\right)$ and using them in the second relation of Eq. (6), the diffusion coefficient ${D}_{j}^{(2)}\left(x,t\right)$ is determined. In addition, the first relation in Eq. (6) gives ${D}_{j}^{(1)}\left(x,t\right)$ by estimating ${M}^{(1)}\left(x,t\right)$ from the data. There are numerous studies regarding the use of the jump-diffusion Eq. (5), which describe the random evolution of neuron dynamics^19,20^, stochastic resonance^21^ and climate data^22^.

### Distinguishing between purely diffusive and jump-diffusion processes

In the sample path of many empirical time series, it is often observed that fluctuations are interrupted by sudden long-amplitude jumps between different states of a system^24^. The studies have shown that empirical detection of jumps is difficult because, in the real world, only discrete data from continuous-time models are available. In general, when data sampled at discrete intervals a sequence of discontinuous jump events will appear in the sampled path, even though the underlying path is continuous. The study on higher-order temporal approximations of KM conditional moments has shown that a finite sampling $\tau$ affects all the KM coefficients^25–27^. Such studies have found that even for diffusive processes, non-vanishing higher-order conditional moments (> 2) can originate from a discrete sampling. Therefore, the Pawula theorem cannot be used to judge whether the given time series falls under the classification of diffusion processes or jump-diffusion processes. This means that when analyzing empirical time series, one cannot be immediately ensure which dynamical Eq. (2) or (3) is appropriate for modeling the corresponding time series, unless one uses the diagnostic criteria presented in this context. For Langevin and Jump-Diffusion dynamics, there are criteria that can be used to check whether a given time series is inherently continuous or discontinuous. Here are two of the widely used criteria:

1- The first criterion for distinguishing between purely diffusive and jump-diffusion processes is the use of the $\frac{{K}^{\left(4\right)}(x,\tau )}{3{(K}^{\left(2\right)}{\left(x,\tau \right))}^{2}}$ ratio. This criterion was introduced by Lehnertz et al*.*^11^. Their results show that this ratio is close to 1 for diffusive processes and for small $\tau$, but for jump-diffusion processes it diverges to 1/τ, namely:

8 $$ \begin{array}{*{20}c} {\frac{{K^{\left( 4 \right)} \left( {x,\tau } \right)}}{{3(K^{\left( 2 \right)} \left( {x,\tau } \right))^{2} }} \approx 1,} & {{\text{diffusive}}} \\ {\frac{{K_{j}^{\left( 4 \right)} \left( {x,\tau } \right)}}{{3(K_{j}^{\left( 2 \right)} \left( {x,\tau } \right))^{2} }}\sim \frac{1}{\tau }} & {{\text{jumpy}}} \\ \end{array} $$

As it can be seen, using ${K}^{(2)}(x,\tau )$ and ${K}^{(4)}(x,\tau )$ will be problematic to detect jumps in the range of small time interval τ. In such a case, the next criterion can be used.

2- The second criterion to distinguish diffusive from jump-diffusion processes is based on the ratio of the fourth- and sixth-order KM conditional moments known as the Q-ratio, which was introduced in the same article^11^ by Lehnertz et al*.* as follows:

$$Q\left(x, \tau \right)=\frac{{K}^{(6)}(x,\tau )}{5{K}^{(4)}(x,\tau )}$$

Using expansion of the KM conditional moments in terms of $\tau$, they found that when the process is purely diffusive $Q\left(x, \tau \right)={D}^{(2)}\left(x\right)\tau$ (Linearly dependent on $\tau$), while when the process has a jumpy behavior, $Q\left(x, \tau \right)={\sigma }_{\xi }^{2}$ (constant and independent of $\tau$), where ${D}^{(2)}(x)$ is the diffusion coefficient, and ${\sigma }_{\xi }^{2}$ is the jump amplitude in jump-diffusion modeling.

In summary, by estimating the following $Q$-ratio from the data, one can be sure which dynamical Equation is appropriate to model the given time series:

9 $$ \begin{array}{*{20}c} {\frac{{K^{\left( 6 \right)} \left( {x,\tau } \right)}}{{5K^{\left( 4 \right)} \left( {x,\tau } \right)}} = D^{\left( 2 \right)} \left( x \right)\tau ,} & {{\text{diffusive}}} \\ {\frac{{K^{\left( 6 \right)} \left( {x,\tau } \right)}}{{5K^{\left( 4 \right)} \left( {x,\tau } \right)}} = \sigma_{\xi }^{2} ,} & {{\text{jumpy}}} \\ \end{array} $$

In the next sections after introducing our dynamical stochastic equation, we will also define a new criterion to differentiate diffusion processes from jump-diffusion processes.

## Introducing the proposed method and results

As mentioned, when analyzing a time series sampled with time intervals $\tau$, successive discontinuities in the sampled path are observed, despite the fact that the underlying path is continuous^11^. In addition, one of the main problems when using data sampled at discrete times is the distinction between discontinuities caused by continuous stochastic processes, and genuine discontinuities in the sample path of time series that were caused by finite sampling of continuous stochastic processes^11^. Some points like this raise the question: *Is it possible to use only jump-drift processes to describe the random evolution of a time series sampled with finite time intervals *$\tau$*?* To address this question, we introduce a new modeling that attributes any stochastic variation in the sample path of a given time series to a jump event, regardless of whether the underlying trajectory is continuous or not. Based on this, we build a new dynamical stochastic equation, and call it the jump-jump equation, which in its general form includes a deterministic drift term and several stochastic terms with jumpy behaviors as follows:

10 $$dx\left(t\right)={D}^{\left(1\right)}\left(x\right)dt+\sum_{i=1}^{N}{\xi }_{i}d{J}_{i}\left(t\right),$$

where ${D}^{\left(1\right)}\left(x\right)$ indicates the deterministic part of the process and ${J}_{1 }\left(t\right),{J}_{2 }\left(t\right), etc$ are Poisson jump processes. The jumps have rates ${\lambda }_{1},{\lambda }_{2 },etc$ and sizes ${\xi }_{1},{\xi }_{2},etc$, which we assume they have zero mean Gaussian distributions with variances (amplitudes)${\sigma }_{\xi 1}^{2},{\sigma }_{\xi 2}^{2}, etc$, respectively. We start with the simplest form of Eq. (10), which includes a drift term and only a jump process. It will be shown that such a jump-drift equation is able to model time series that are classified as continuous processes. Afterwards, we extend modeling by considering more jump processes in Eq. (10), and use it to model time series with more varied amplitudes. In each step, we will demonstrate that all unknown coefficients and functions involved in this model can be derived directly from the measured time series data.

### Jump-drift modeling

We now consider Eq. (10) with a drift term and a jump process (a jump-drift equation), and show that it can be used to model time series belonging to the class of continuous processes when the data are sampled at discrete intervals. The general form of a jump-drift equation is as follows:

11 $$dx\left(t\right)={D}^{\left(1\right)}\left(x,t\right)dt+\xi dJ\left(t\right),$$

where ${D}^{\left(1\right)}\left(x,t\right)$ denotes the drift part of the process, and $J(t)$ is a Poisson jump process characterized by the rate $\lambda (x,t)$ and the size $\xi$. We assume that $\xi$ is a random variable, and has a zero mean Gaussian distribution, i.e. $\xi \sim N(0, {\sigma }_{\xi }^{2})$. The variance of this distribution (${\sigma }_{\xi }^{2}$) is called the jump amplitude, and in general may depend on $x$ and $t$. We will show that all unknown parameters and functions required in this modeling can be estimated based on a data-driven approach from measured time series. Before doing so, it is necessary to mention two points:

1) We assume the case that $J(t)$ is a homogeneous Poisson jump process with a constant jump rate $\lambda$. The jump rate represents the expected number of jumps that will occur per unit time. It follows that the number of jumps occurring in the interval of $(t, t+dt]$ follows a Poisson distribution with the associated parameter $\lambda dt$. On the other hand, a jump event has two states of occurrence 1 and non-occurrence 0, of which only one will occur in each infinitesimal $dt$. The last point shows that in the Poisson process, the occurrence of an event in each small interval of time is defined as a Bernoulli variable. That is, $dJ$ takes only the values 1 and 0 with probabilities $\lambda dt$ and $1-\lambda dt$, respectively.

2) Up to the first orders in $dt,$ the statistical moments of $dJ$ are given by the following relation^8,10^:

$$\left\langle{{\left(dJ\right)}^{m}}\right\rangle = \lambda dt$$

With these two points in mind, we now present a data-driven approach to estimate the drift and jump properties required in this modeling. This method can be used for both stationary and non-stationary time series, and the results are applicable to both.

### Non-parametric estimation of jump-drift processes.

#### Theorem 1

For a jump-drift process described by the dynamical Eq. (11), all the functions and parameters required to model the process can be estimated non-parametrically by estimating KM coefficients from measured time series as follows:

12 $$ \begin{gathered} M^{\left( 1 \right)} \left( {x,t} \right) = D^{\left( 1 \right)} \left( {x,t} \right) \hfill \\ M^{\left( n \right)} \left( {x,t} \right) = \lambda \left( {x,t} \right)\left\langle {\xi^{n} } \right\rangle \,{\text{for}}\, n \ge 2 \hfill \\ \end{gathered} $$

We have provided a proof for this theorem in the appendix. For non-stationary processes, all functions and parameters are time-dependent, but in the following, we focus on stationary processes, and omit the t-dependence in Eq. (12) to improve readability.

We can estimate the drift function ${D}^{\left(1\right)}\left(x\right)$ using the first relation in (12). The jump amplitude ${\sigma }_{\xi }^{2}(x)$ and the jump rate $\lambda (x)$ can be estimated using the relation $\left\langle{{\xi }^{2l}}\right\rangle =\frac{2l!}{{2}^{l}l!}\left\langle{{\xi }^{2}}\right\rangle^{l}$ for the Gaussian random variable $\xi$ in the last relation in Eq. (12) with $n=2$ and $n=4$. Therefore, we have:

13 $$ \begin{gathered} D^{\left( 1 \right)} \left( x \right) = M^{\left( 1 \right)} \left( x \right) \hfill \\ \sigma_{\xi }^{2} \left( x \right) = \frac{{M^{\left( 4 \right)} \left( x \right)}}{{3M^{\left( 2 \right)} \left( x \right)}} \hfill \\ \lambda \left( x \right) = \frac{{M^{\left( 2 \right)} \left( x \right)}}{{\sigma_{\xi }^{2} \left( x \right)}}, \hfill \\ \end{gathered} $$

where $${M}^{(n)}\left(x\right)= \underset{dt\to 0}{{\text{lim}}}\frac{1}{dt}{K}^{\left(n\right)}\left(x\right).$$

We now argue that if Eq. (11) is able to describe the random evolution of a sampled time series $x(t)$ belonging to the class of diffusion processes, then the following conditions should be held:

1. The last relation in (12) in terms of conditional moments ${K}^{\left(n\right)}\left(x\right)$ is written as follows:
  $${K}^{\left(n\right)}\left(x\right)=\left\langle{{\xi }^{n}}\right\rangle\lambda \left(x\right)dt,$$
  where with $n=2$ and $n=4$ it leads to:
  14 $$ \begin{gathered} K^{\left( 2 \right)} \left( x \right) = \sigma_{\xi }^{2} \left( x \right)\lambda \left( x \right)dt \hfill \\ K^{\left( 4 \right)} \left( x \right) = 3\left( {\sigma_{\xi }^{2} \left( x \right)} \right)^{2} \lambda \left( x \right)dt, \hfill \\ \end{gathered} $$
  Extracting the ratio $\frac{{K}^{(4)}(x)}{3{(K}^{\left(2\right)}{\left(x\right))}^{2}}$ from these relations leads to:
  $$\frac{{K}^{\left(4\right)}(x)}{3{(K}^{\left(2\right)}{\left(x\right))}^{2}}=\frac{1}{\lambda \left(x\right)dt}$$
  On the other hand, we know from Eq. (8) that this ratio is approximately equal to 1 in diffusion processes for small $dt$, as a result:
  $$\lambda \left(x\right)dt\approx 1$$
  This criterion can be used as a possibility for numerical verification of Pawula theorem. Employing this measure, one can ensure that the given time series belongs to the class of diffusive processes or not:
  15 $$ \begin{gathered} \lambda \left( x \right)dt = 1,\,\, {\text{diffusive}} \hfill \\ \lambda \left( x \right)dt \ne 1,\,\, {\text{jumpy}} \hfill \\ \end{gathered} $$
2. Comparing ${K}^{\left(2\right)}\left(x\right)$ presented in (14) with the second-order conditional moment used in the Langevin modeling i.e.${K}^{\left(2\right)}\left(x\right)={D}^{\left(2\right)}\left(x\right) dt$, we obtain:
  $${\sigma }_{\xi }^{2}\left(x\right)\lambda \left(x\right)dt={D}^{\left(2\right)}\left(x\right)dt$$
  Applying the condition $\lambda \left(x\right)dt=1$ for diffusion processes leads to the following result:
  16 $${\sigma }_{\xi }^{2}\left(x\right)={D}^{\left(2\right)}\left(x\right) dt$$
  This means that if we use the drift-jump Eq. (11) to model diffusion processes, then the estimation of the jump amplitude ${\sigma }_{\xi }^{2}\left(x\right)$ will lead to the estimation of the diffusion coefficient ${D}^{\left(2\right)}\left(x\right)$ required in Langevin modeling.

In order to test the validity of the proposed modeling, we reconstructed a diffusion process with preset drift and diffusion coefficients using a synthetic time series sampled with time intervals $dt$. Diffusive process generated using the discretization of Eq. (3) in Euler–Maruyama scheme [28] with a sampling interval $dt=0.001$ and with functions ${D}^{\left(1\right)}\left(x\right)=-x$ and ${D}^{\left(2\right)}\left(x\right)=1$ (the Ornstein–Uhlenbeck process).

**Figure 1:**
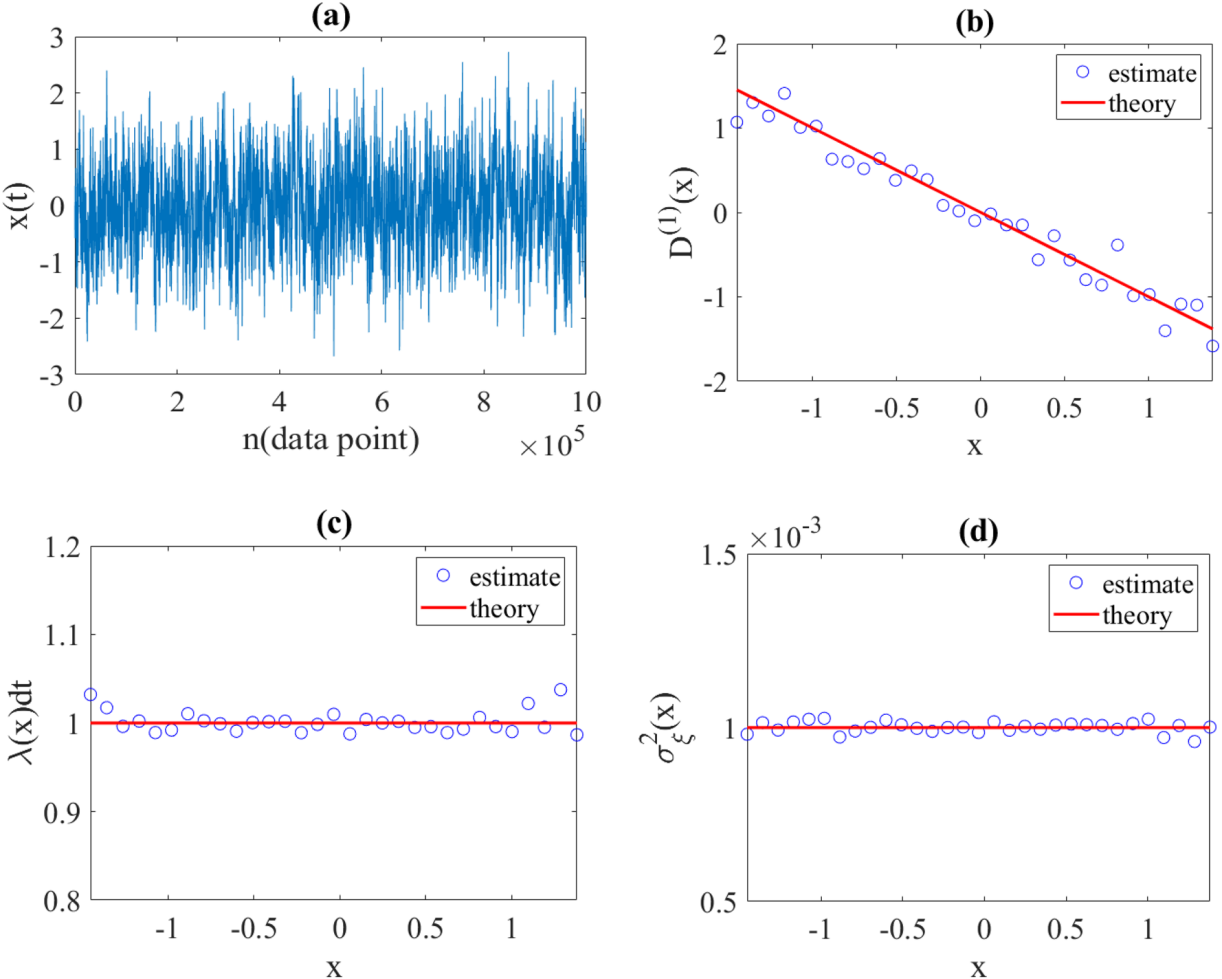
(**a**) Synthetic time series (${10}^{6}$ data points) generated by Langevin equation with functions ${{\varvec{D}}}^{\left(1\right)}\left({\varvec{x}}\right)=-{\varvec{x}}$ and ${{\varvec{D}}}^{\left(2\right)}\left({\varvec{x}}\right)=1$ and with a time interval ${\varvec{d}}{\varvec{t}}=0.001$. (**b**) Estimated drift, (**c**) estimated jump rate, and (**d**) estimated jump amplitude obtained using proposed jump-drift modeling. As can be seen ${\varvec{\lambda}}\left({\varvec{x}}\right){\varvec{d}}{\varvec{t}}\approx 1$ and ${{\varvec{\sigma}}}_{{\varvec{\xi}}}^{2}\left({\varvec{x}}\right)\approx {{\varvec{D}}}^{\left(2\right)}\left({\varvec{x}}\right){\varvec{d}}{\varvec{t}}$, which confirms correctnes of Eqs. (15) and (16).

Afterwards, the unknown parameters ${D}^{\left(1\right)}\left(x\right)$,$\lambda \left(x\right)$ and ${\sigma }_{\xi }^{2}\left(x\right)$ required for jump-drift modeling were estimated using the relations in (13). As explained, we expected $\lambda \left(x\right)dt\approx 1$ and ${\sigma }_{\xi }^{2}\left(x\right)={D}^{\left(2\right)}\left(x\right)dt=0.001$, which the obtained results were confirmed (see Fig. 1).

**Figure 2:**
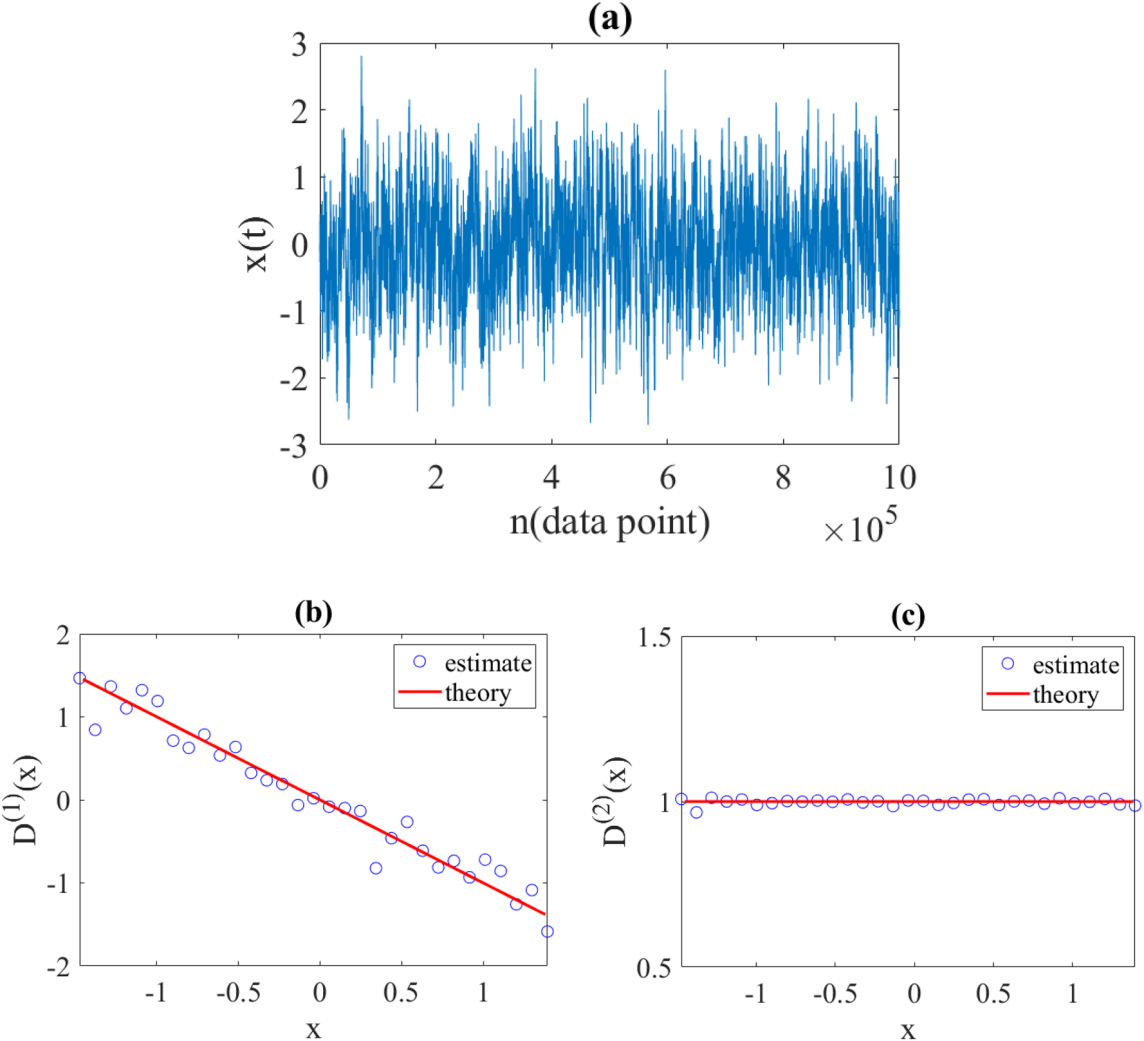
(**a**) Reconstructed data by jump-drift Eq. (11) using estimated parameters from Fig. 1 with time interval ∆t = 0.001. (**b**) Estimated drift coefficient, and (**c**) estimated diffusion coefficient, obtained from reconstructed data. The red lines are the initial coefficients. As can be seen, the good agreement between the estimated coefficients, and the original coefficients confirms that the jump-drift equation is able to describe the discrete time evolution of a diffusion process.

Furthermore, to ensure that jump-drift modeling is capable to reconstruct a time series for $x(t)$ that is statistically similar to the original diffusion time series, we reconstructed a data set by applying the obtained parameters to the jump-drift equation Eq. (11). Afterwards, ${D}^{\left(1\right)}\left(x\right)$ and ${D}^{\left(2\right)}\left(x\right)$ were estimated from the reconstructed data, and we found a very good agreement between these estimated coefficients and the corresponding original ones (see Fig. 2).

### Jump-jump modeling

In this section, we expand the jump-drift dynamical Eq. (11), and do not limit it to only a jump process. We begin by considering two jump processes with two different amplitudes in Eq. (10). Before continuing the discussion, let us explain how the idea of ​​including these two jump processes comes about.

The jump-diffusion Eq. (5) that is able to construct a trajectory with jump discontinuities consists a deterministic drift term and two stochastic terms with diffusive and jumpy behaviors. On the other hand, when data sampled at discrete time intervals from a jump-diffusion process, two types of discontinuities are observed in the path of the sampled time series. Those discontinuities that originate from finite sampling of the diffusive part of the process, and have a smaller amplitude, and those discontinuities that arise from genuine jump events and have a larger amplitude. Based on this, we build a new equation including a deterministic drift term and two stochastic terms with jumpy behavior. The aim of this article is to introduce this jump-jump equation, which enables us to generate sample paths with successive discontinuities, but with two different distributed sizes. A jump-jump equation is as follows:

17 $$dx\left(t\right)={D}^{\left(1\right)}\left(x,t\right)dt+{\xi }_{1}d{J}_{1}\left(t\right)+{\xi }_{2}d{J}_{2}\left(t\right),$$

where ${D}^{\left(1\right)}\left(x,t\right)$ indicates the deterministic part of the process and ${J}_{1 }\left(t\right)$ and ${J}_{2 }(t)$ are Poisson jump processes. The jumps have rates ${\lambda }_{1}$ and ${\lambda }_{2}$, and sizes ${\xi }_{1}$ and ${\xi }_{2}$, which we assume have zero mean Gaussian distributions with variances ${\sigma }_{\xi 1}^{2}$ and ${\sigma }_{\xi 2}^{2}$, respectively (or any symmetric distribution with finite statistical moments). In general, the jump rates ${\lambda }_{1}$ and ${\lambda }_{2}$ and statistical moments of ${\sigma }_{\xi 1}^{2}$ and ${\sigma }_{\xi 2}^{2}$ may be functions of state variable $x$ and time $t$. We also assume that any discontinuity in the sample path is caused by the occurrence of only one of the jump events $d{J}_{1}\left(t\right)$ or $d{J}_{2}\left(t\right)$, and two jumps do not occur simultaneously. The meaning of this condition is that in the time interval $(t,t+dt]$, if for example $d{J}_{1}\left(t\right)$ occur, and takes the value 1, then $d{J}_{2}\left(t\right)$ does not occur, and its value becomes zero and vice versa. Applying this condition enables us to construct a time series via Eq. (17) whose corresponding trajectory consists of successive jump discontinuities with different amplitudes and jump rates. In other words, by applying this condition, Eq. (17) is able to describe the random evolution of a jump-jump process, a process whose corresponding time series consists of the union of two data sets belonging to two jump processes with different amplitudes and rates. We now discuss a nonparametric approach to estimating drift and jump characteristics directly from the measured time series data. This method can be applied to both stationary and non-stationary time series, and the results can be applied to both.

### Non-parametric estimation of jump-jump processes

#### Theorem 2

For a jump-jump process described by the dynamical Eq. (17), all the functions and parameters required to model the process can be estimated non-parametrically by estimating KM coefficients from measured time series as follows:

18 $$ \begin{gathered} M^{\left( 1 \right)} \left( {x,t} \right) = D^{\left( 1 \right)} \left( {x,t} \right) \hfill \\ M^{\left( n \right)} \left( {x,t} \right) = \left\langle {\xi_{1}^{n} } \right\rangle {\uplambda }_{1} \left( {x,t} \right) + \left\langle {\xi_{2}^{n} } \right\rangle {\uplambda }_{2} \left( {x,t} \right) for n \ge 2 \hfill \\ \end{gathered} $$

In the Appendix, we have presented a proof for this theorem. In this section, as before, we focus on stationary processes, and we remove the t-dependencies in Eq. (18).

The five unknown parameters required for this modeling are ${D}^{\left(1\right)}\left(x\right),$ ${\uplambda }_{1}\left(x\right),$ ${\uplambda }_{2}\left(x\right),$ ${\sigma }_{\xi 1}^{2}\left(x\right)$ and ${\sigma }_{\xi 2}^{2}\left(x\right)$. The first relation in this theorem gives us the estimate for the drift coefficient, which is equal to the first-order KM coefficient, namely:

19 $${D}^{\left(1\right)}\left(x\right)={M}^{\left(1\right)}\left(x\right)$$

Additionally, from the last relation in Eq. (18) for $n=2, 4, 6, 8$, we can derive a system of equations to estimate the parameters of jump processes as follows (we use the relation $\left\langle{{\xi }^{2l}}\right\rangle =\frac{\left(2l\right)!}{{2}^{l}l!}\left\langle{{\xi }^{2}}\right\rangle^{l}$ for the Gaussian random variables ${\xi }_{1}$ and ${\xi }_{2})$:

20 $$ \begin{gathered} M^{\left( 2 \right)} \left( x \right) = \sigma_{\xi 1}^{2} \left( x \right){\uplambda }_{1} \left( x \right) + \sigma_{\xi 2}^{2} \left( x \right){\uplambda }_{2} \left( x \right) \hfill \\ M^{\left( 4 \right)} \left( x \right) = 3\left( {\sigma_{\xi 1}^{2} \left( x \right)} \right)^{2} {\uplambda }_{1} \left( x \right) + 3\left( {\sigma_{\xi 2}^{2} \left( x \right)} \right)^{2} {\uplambda }_{2} \left( x \right) \hfill \\ M^{\left( 6 \right)} \left( x \right) = 15\left( {\sigma_{\xi 1}^{2} \left( x \right)} \right)^{3} {\uplambda }_{1} \left( x \right) + 15\left( {\sigma_{\xi 2}^{2} \left( x \right)} \right)^{3} {\uplambda }_{2} \left( x \right) \hfill \\ M^{\left( 8 \right)} \left( x \right) = 105\left( {\sigma_{\xi 1}^{2} \left( x \right)} \right)^{4} {\uplambda }_{1} \left( x \right) + 105\left( {\sigma_{\xi 2}^{2} \left( x \right)} \right)^{4} {\uplambda }_{2} \left( x \right) \hfill \\ \end{gathered} $$

By solving this system of nonlinear equations, the unknowns ${\lambda }_{1}(x),{\lambda }_{2 }(x),{\sigma }_{\xi 1}^{2}(x),{\sigma }_{\xi 2}^{2}(x)$ are estimated using ${M}^{\left(2\right)}\left(x\right),{M}^{\left(4\right)}\left(x\right)$ and ${M}^{\left(6\right)}\left(x\right)$, which are obtained from the data. Since the parametric solution of this system of equations leads to long and boring relations, we refrain from presenting them, and use the numerical methods.

**Figure 3:**
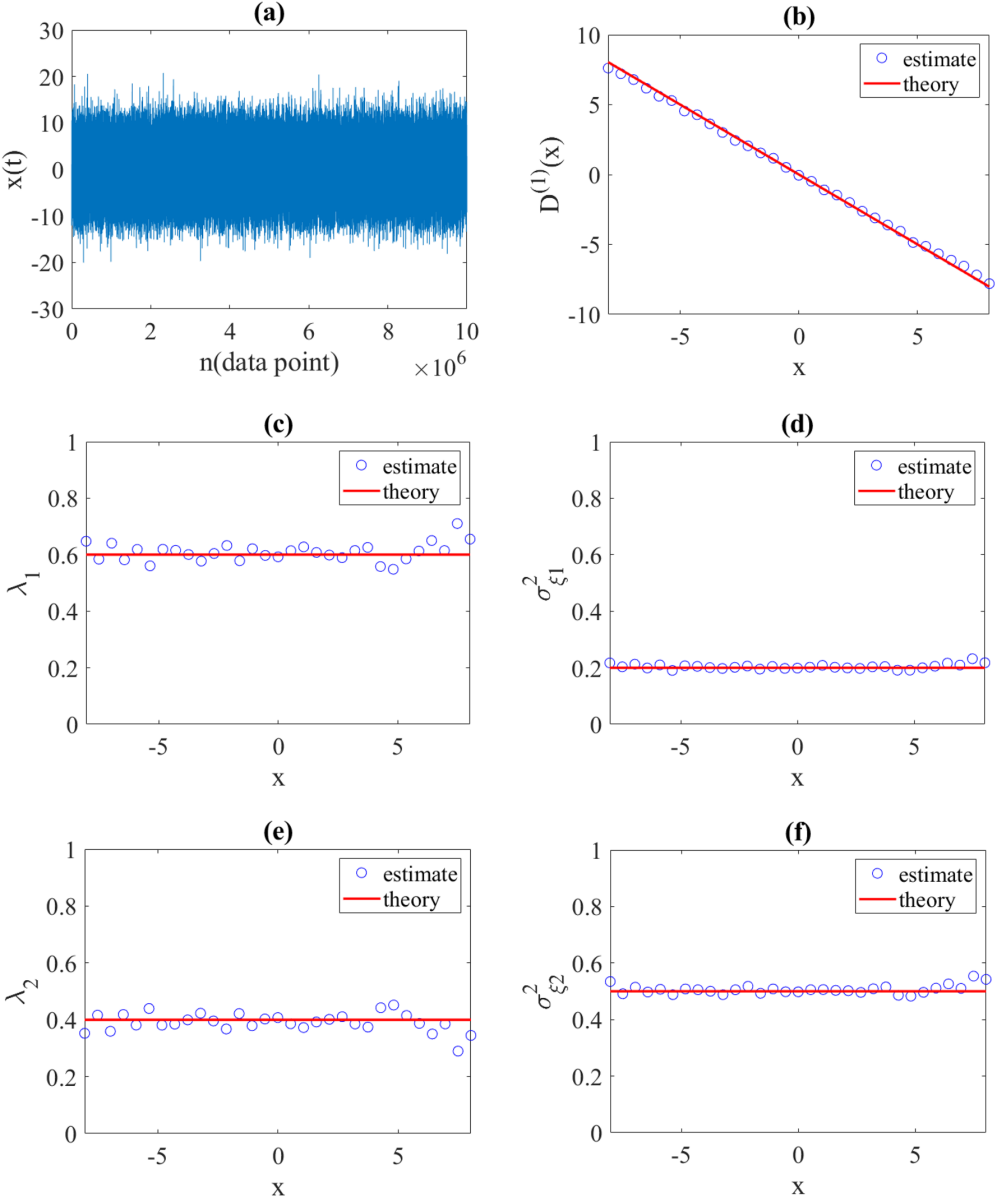
(**a**) Illustration of a synthetic time series generated using the proposed jump-jump Eq. (17) with a time interval $\Delta {\varvec{t}}=0.01$, a drift function ${{\varvec{D}}}^{\left(1\right)}\left({\varvec{x}}\right)=-{\varvec{x}}$ and two constant jump amplitudes ${{\varvec{\sigma}}}_{{\varvec{\xi}}1}^{2}\left({\varvec{x}}\right)=0.2$ and ${{\varvec{\sigma}}}_{{\varvec{\xi}}2}^{2}\left({\varvec{x}}\right)=0.5$ with jump rates ${{\varvec{\lambda}}}_{1}\left({\varvec{x}}\right)=0.6$ and ${{\varvec{\lambda}}}_{2}\left({\varvec{x}}\right)=0.4$, respectively. (**b**) Estimated drift term and (**c**–**f**) estimated jumps characteristics using relations in Eq. (20). The red lines are the corresponding theoretical coefficients.

To demonstrate the validity of our approach, we estimated drift and jumps characteristics from synthetic time series generated with preset coefficients. First, we considered Eq. (17) with $${D}^{\left(1\right)}\left(x\right)=-x$$ as a linear drift function and two constant jump amplitudes $${\sigma }_{\xi 1}^{2}\left(x\right)=0.2$$ and $${\sigma }_{\xi 2}^{2}\left(x\right)=0.5$$ with constant jump rates per data point $${\uplambda }_{1}\left(x\right)=0.6$$ and $${\uplambda }_{2}\left(x\right)=0.4$$, respectively. It is worth noting that the jump rate per data point is different from the jump rate per unit of time in a $$dt$$, i.e.$$\lambda (per data point)=\lambda \left(per unit of time\right)*dt$$. We generated synthetic time series by discretizing Eq. (17) using Euler–Maruyama discretization scheme with $$dt=0.01$$. Afterwards, we estimated the drift function and jump characteristics from the synthetic time series using relations present in (19) and (20). Very good agreement was observed between all estimates and initial functions and parameters (see Fig. 3).

**Figure 4:**
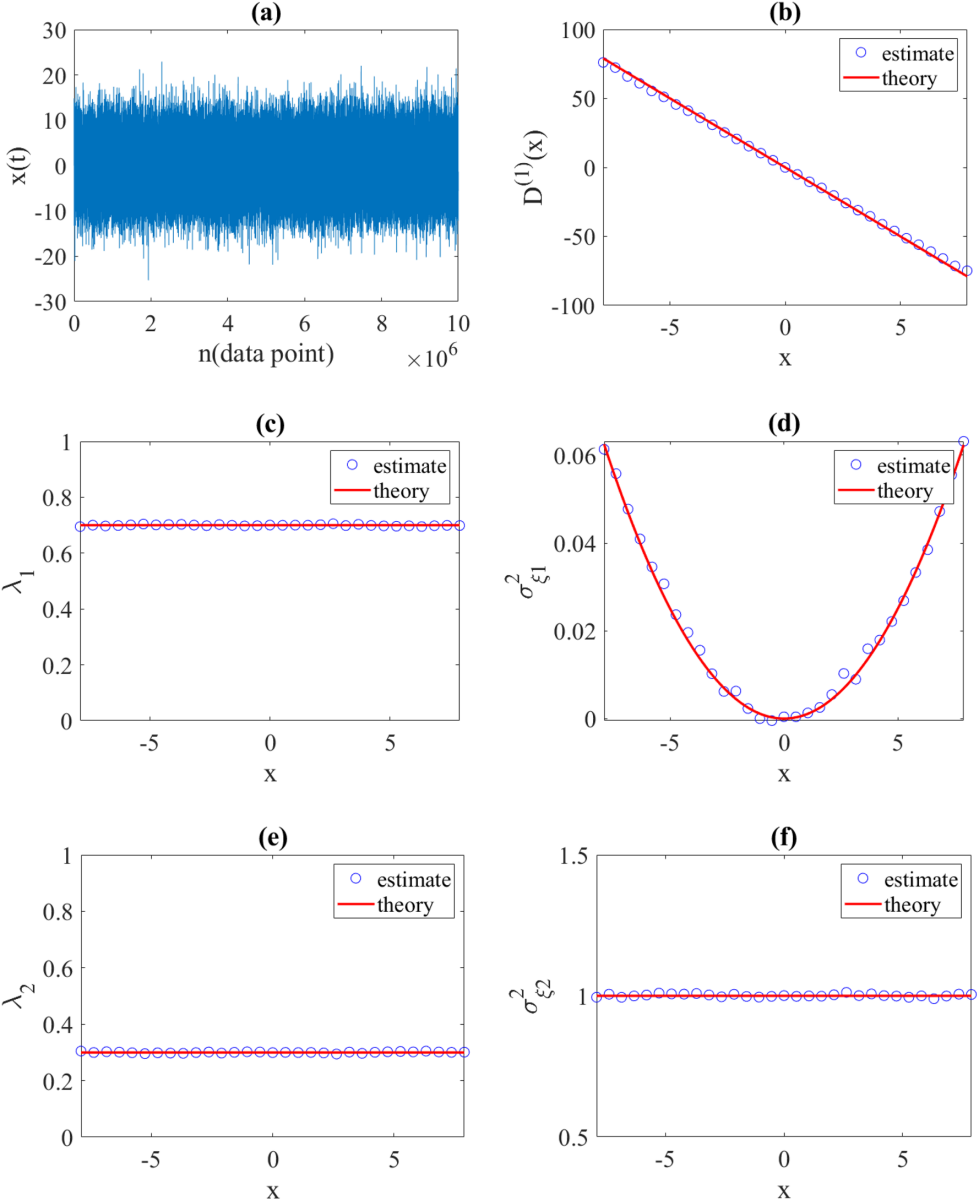
(**a**) Illustration of a synthetic time series generated using the proposed jump-jump Eq. (17) with a time interval $\Delta t=0.001$, a linear drift ${D}^{\left(1\right)}\left(x\right)=-10x$ and two jump amplitudes ${\sigma }_{\xi 1}^{2}\left(x\right)=0.001{x}^{2}$ and ${\sigma }_{\xi 2}^{2}\left(x\right)=1$ with jump rates ${\lambda }_{1}\left(x\right)=0.7$ and ${\lambda }_{2}\left(x\right)=0.3$, respectively. (**b**) Estimated drift coefficient. (**c**–**f**) Estimated jump characteristics using relations in (20). The red lines are the corresponding theoretical coefficients.

As a second example, we considered Eq. (17) with a linear drift function $${D}^{\left(1\right)}\left(x\right)=-10x$$ and two jump amplitude as $${\sigma }_{\xi 1}^{2}\left(x\right)=b{x}^{2}$$ ($$b=0.001)$$ and $${\sigma }_{\xi 2}^{2}\left(x\right)=1$$, with constant jump rates per data point $${\uplambda }_{1}\left(x\right)=0.7$$ and $${\uplambda }_{2}\left(x\right)=0.3$$, respectively. We proceeded as before, and generated an exemplary synthetic time series using the discretization of Eq. (17) in Euler–Maruyama scheme with a sampling interval $$dt=0.001$$. Again, a very good agreement was found between the estimated and predetermined functions and parameters (see Fig. 4).

### Jump-jump modeling with constant coefficients and parameters

Because of its practical uses, in this section we focus on a special case of Eq. (17), where all coefficients and parameters are assumed constant and none of them are time-dependent or state-dependent. For this purpose, we rewrite the Eq. (17) as follows:

21 $$dx\left(t\right)=\mu dt+{\xi }_{1}d{J}_{1}\left(t\right)+{\xi }_{2}d{J}_{2}\left(t\right),$$

where μ is the drift parameter and other parameters are the same as previously defined. Similar to the proof provided in Theorem 2, one can prove that all necessary parameters and coefficients in this modeling are obtained non-parametrically by estimating the statistical moments of the increments of the measured time series as follows:

22 $$ \begin{gathered} M_{1} = \mu \hfill \\ M_{n} = \left\langle {\xi_{1}^{n} } \right\rangle {\uplambda }_{1} + \left\langle {\xi_{2}^{n} } \right\rangle {\uplambda }_{2} {\text{for }}n \ge 2, \hfill \\ \end{gathered} $$

where ${M}_{n}= \underset{dt\to 0}{{\text{lim}}}\frac{1}{dt}\left\langle{d{x}^{n}}\right\rangle$ are the statistical moments of the increments of the time series, namely $dx=x\left(t+dt\right)-x\left(t\right).$ As before, we derive the following relations from Eq. (22):

23 $$ \begin{gathered} M_{1} = \mu \hfill \\ M_{2} = \sigma_{\xi 1}^{2} {\uplambda }_{1} + \sigma_{\xi 2}^{2} {\uplambda }_{2} \hfill \\ M_{4} = 3\left( {\sigma_{\xi 1}^{2} } \right)^{2} {\uplambda }_{1} + 3\left( {\sigma_{\xi 2}^{2} } \right)^{2} {\uplambda }_{2} \hfill \\ M_{6} = 15\left( {\sigma_{\xi 1}^{2} } \right)^{3} {\uplambda }_{1} + 15\left( {\sigma_{\xi 2}^{2} } \right)^{3} {\uplambda }_{2} \hfill \\ M_{8} = 105\left( {\sigma_{\xi 1}^{2} } \right)^{4} {\uplambda }_{1} + 105\left( {\sigma_{\xi 2}^{2} } \right)^{4} { \lambda }_{2} \hfill \\ \end{gathered} $$

By solving this system of equations, the 5 unknown parameters, i.e. $\mu$ and ${\lambda }_{1},{\lambda }_{2 },{\sigma }_{\xi 1}^{2},{\sigma }_{\xi 2}^{2}$ can be obtained.

**Figure 5:**
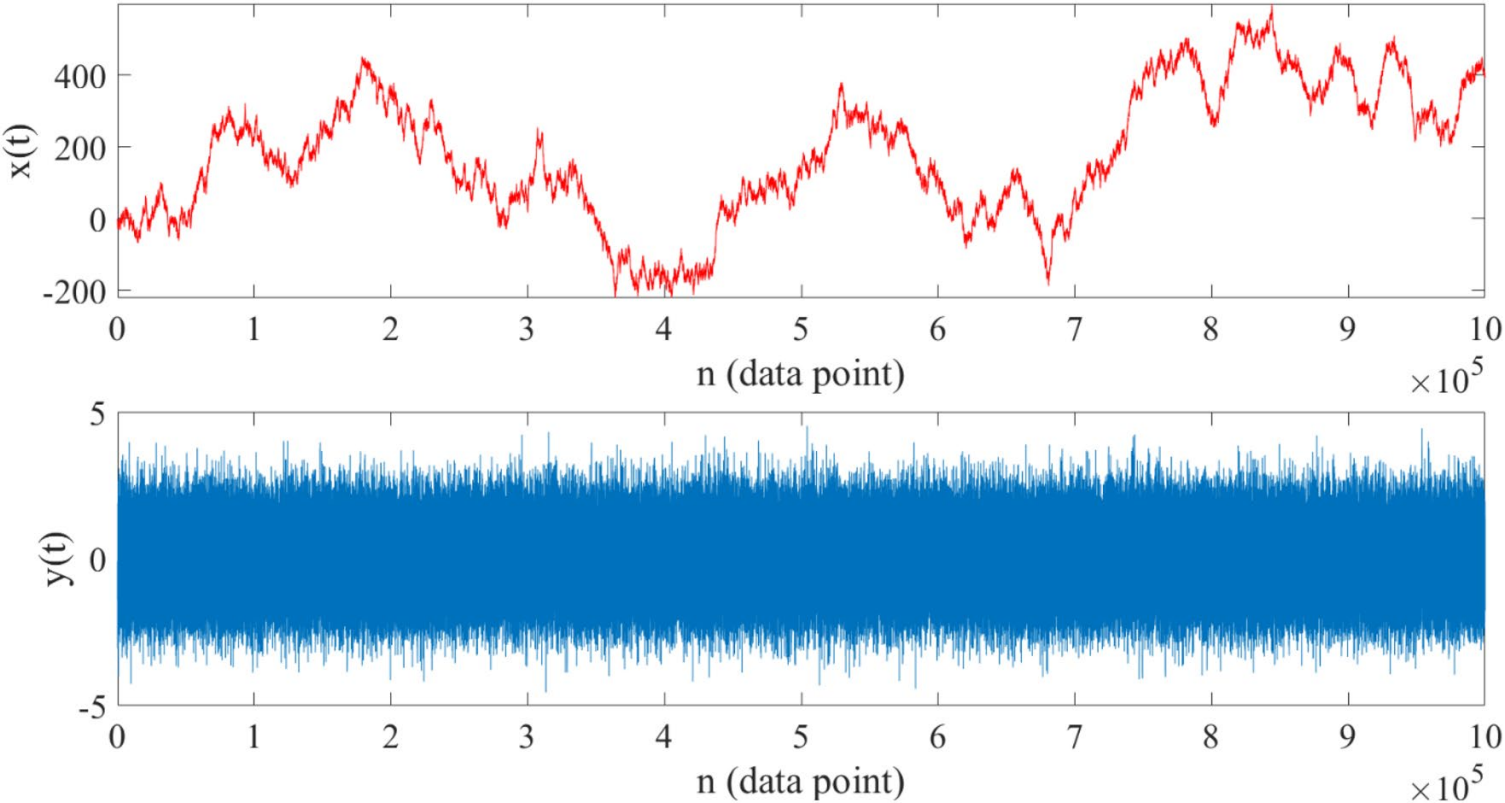
Upper panel: ${10}^{6}$-step random walk generated using Eq. (21) with constant drift parameter ${\varvec{\mu}}=1$ and constant jump components ${{\varvec{\upsigma}}}_{{\varvec{\upxi}}1}^{2}=1$,${{\varvec{\sigma}}}_{{\varvec{\xi}}2}^{2}=0.3$ and ${{\varvec{\uplambda}}}_{1}=0.4$, ${{\varvec{\lambda}}}_{2}=0.6$ with a time interval $\Delta {\varvec{t}}=0.001$ (starting from zero). Lower panel: increments of the generated time series ${\varvec{y}}\left({\varvec{t}}\right)={\varvec{x}}\left({\varvec{t}}+\Delta {\varvec{t}}\right)-{\varvec{x}}\left({\varvec{t}}\right)$.

Again, to investigate the validity of this approach, we estimated these parameters from synthetic time series generated with known drift and jump parameters. We considered Eq. (21) with $\mu =1$ and two constant jump amplitudes ${\sigma }_{\xi 1}^{2}=1$ and ${\sigma }_{\xi 2}^{2}=0.3$ with two constant jump rates per data point ${\lambda }_{1}=0.4$ and ${\lambda }_{2}=0.6$, respectively. We generated synthetic time series $x\left(t\right)$ using the Euler–Maruyama scheme with a sampling interval $dt=0.001$. A sample path of $x\left(t\right)$ is shown in Fig. 5. In addition, we constructed a new time series $y(t)$ based on the increments of $x(t)$, i.e.$y\left(t\right)=x\left(t+\Delta t\right)-x\left(t\right)$ (the trajectory of $y(t)$ is also shown in Fig. 5).

By calculating the statistical moments of $y(t)$ for $n=1, 2, 4, 6, 8$ and substituting in Eqs. (23), and then solving this system of equations, the following results were estimated, which are in very good agreement with the original values:

$$\mu \approx 1.04$$, $${\sigma }_{\xi 1}^{2}\approx 1.0002$$ , $${\sigma }_{\xi 2}^{2}\approx 0.3004$$ , $${\uplambda }_{1}\approx 0.3992$$,$${\uplambda }_{2}\approx 0.6007$$

### Expansion of the jump-jump equation

The strength of jump-jump modeling is that if the amplitude of fluctuations in a given time series is so diverse that its random evolution cannot be described using only two jump processes such as seen in Eq. (17) or (21). Afterwards, the stochastic part of the Eq. (10) can be expanded by considering more jump processes. For example, Eq. (21) is expanded as follows considering three jump processes:

24 $$dx\left(t\right)=\mu dt+{\xi }_{1}d{J}_{1}\left(t\right)+{\xi }_{2}d{J}_{2}\left(t\right)+{\xi }_{3}d{J}_{3}(t)$$

As before, we assume that any random variation in the time series data is due to the occurrence of only one of the jump events, and that two or more jump events do not occur simultaneously. That is, when in a time step $d{J}_{1}\left(t\right)$ occur and takes the value 1,$d{J}_{2}\left(t\right)$ and $d{J}_{3}\left(t\right)$ do not occur and their values are zero, and so on. The following section discusses a nonparametric approach to estimate the drift parameter $\mu$ and the jump characteristic ${\lambda }_{1},{\lambda }_{2 },{\lambda }_{3},{\sigma }_{\xi 1}^{2},{\sigma }_{\xi 2}^{2},{\sigma }_{\xi 3}^{2}$ required in this modeling.

### Theorem 3: parametric estimation of jump-jump processes

#### Theorem 3

For a jump-jump process described by the dynamical Eq. (24), all the functions and parameters required to model the process can be estimated non-parametrically by estimating KM coefficients from measured time series as follows:

25 $$ \begin{gathered} M_{1} = \mu \hfill \\ M_{n} = \left\langle {\xi_{1}^{n} } \right\rangle {\uplambda }_{1} + \left\langle {\xi_{2}^{n} } \right\rangle {\uplambda }_{2} + \left\langle {\xi_{3}^{n} } \right\rangle {\uplambda }_{3} {\text{for }}n \ge 2, \hfill \\ \end{gathered} $$

where all parameters and coefficients are the same as previously defined. In the Appendix, we have presented a proof for this theorem. As before, the first relation in (25) use for estimating the drift coefficient, which is equal to the first-order KM coefficient:

26 $$\mu ={M}_{1}$$

On the other hand, using the last relation in Eq. (25), with $n=2, 4, 6, 8, 10, 12$, and using the relation $\left\langle{{\xi }^{2l}}\right\rangle =\frac{2l!}{{2}^{l}l!}\left\langle{{\xi }^{2}}\right\rangle^{l}$ for the Gaussian random variables ${\xi }_{1,} {\xi }_{2}$ and ${\xi }_{3}$, one can estimate the 6 unknown parameters ${\lambda }_{1},{\lambda }_{2 },{\lambda }_{3},{\sigma }_{\xi 1}^{2},{\sigma }_{\xi 2}^{2},{\sigma }_{\xi 3}^{2}$ by solving the following system of equations:

27 $$ \begin{gathered} M_{2} = \sigma_{\xi 1}^{2} {\uplambda }_{1} + \sigma_{\xi 2}^{2} {\uplambda }_{2} + \sigma_{\xi 3}^{2} {\uplambda }_{3} \hfill \\ M_{4} = 3\left( {\sigma_{\xi 1}^{2} } \right)^{2} {\uplambda }_{1} + 3\left( {\sigma_{\xi 2}^{2} } \right)^{2} {\uplambda }_{2} + 3\left( {\sigma_{\xi 3}^{2} } \right)^{2} {\uplambda }_{3} \hfill \\ M_{6} = 15\left( {\sigma_{\xi 1}^{2} } \right)^{3} {\uplambda }_{1} + 15\left( {\sigma_{\xi 2}^{2} } \right)^{3} {\uplambda }_{2} + 15\left( {\sigma_{\xi 3}^{2} } \right)^{3} {\uplambda }_{3} \hfill \\ M_{8} = 105\left( {\sigma_{\xi 1}^{2} } \right)^{4} {\uplambda }_{1} + 105\left( {\sigma_{\xi 2}^{2} } \right)^{4} {\uplambda }_{2} + 105\left( {\sigma_{\xi 3}^{2} } \right)^{4} {\uplambda }_{3} \hfill \\ M_{10} = 945\left( {\sigma_{\xi 1}^{2} } \right)^{5} {\uplambda }_{1} + 945\left( {\sigma_{\xi 2}^{2} } \right)^{5} {\uplambda }_{2} + 945\left( {\sigma_{\xi 3}^{2} } \right)^{5} {\uplambda }_{3} \hfill \\ M_{12} = 10395\left( {\sigma_{\xi 1}^{2} } \right)^{6} {\uplambda }_{1} + 10395\left( {\sigma_{\xi 2}^{2} } \right)^{6} {\uplambda }_{2} + 10395\left( {\sigma_{\xi 3}^{2} } \right)^{6} { \lambda }_{3} \hfill \\ \end{gathered} $$

**Figure 6:**
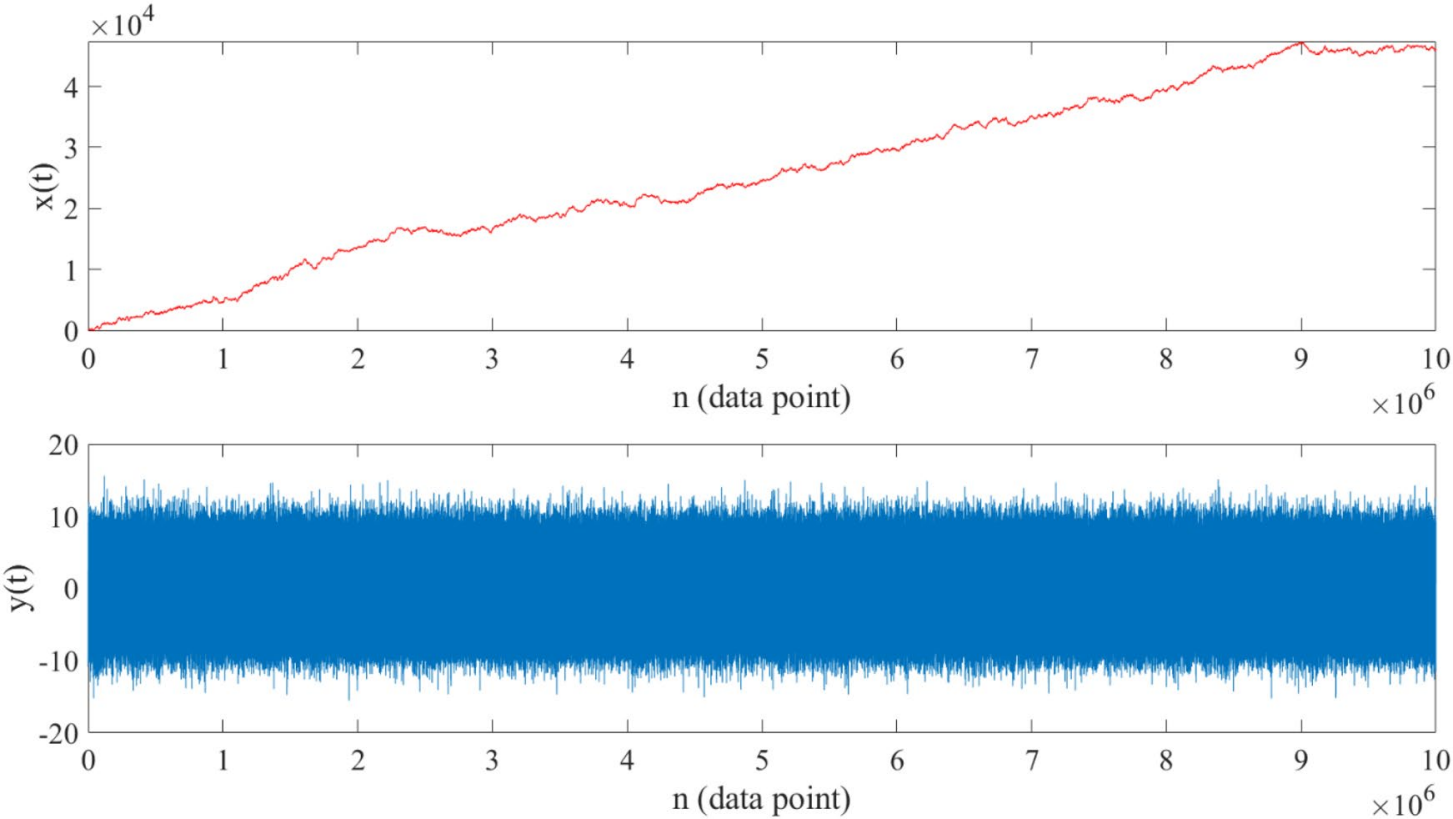
Upper panel: ${10}^{7}$-step random walk generated using Eq. (24) with constant drift parameter ${\varvec{\mu}}=5$ and constant jump components ${{\varvec{\sigma}}}_{{\varvec{\xi}}1}^{2}=0.2$,${{\varvec{\sigma}}}_{{\varvec{\xi}}2}^{2}=0.6$,${{\varvec{\sigma}}}_{{\varvec{\xi}}3}^{2}=10$ and ${{\varvec{\lambda}}}_{1}=0.3$,${{\varvec{\lambda}}}_{2}=0.2$, ${{\varvec{\lambda}}}_{3}=0.5$ with a time interval $\Delta {\varvec{t}}=0.001$ (starting from zero). Lower panel: increments of the generated time series ${\varvec{y}}\left({\varvec{t}}\right)={\varvec{x}}\left({\varvec{t}}+\Delta {\varvec{t}}\right)-{\varvec{x}}\left({\varvec{t}}\right)$.

To demonstrate the validity of this modeling we constructed a synthetic time series $x(t)$ with a constant drift parameter $\mu =5$ and jump amplitudes ${\sigma }_{\xi 1}^{2}=0.2$ and ${\sigma }_{\xi 2}^{2}=0.6$ and ${\sigma }_{\xi 3}^{2}=10$ with constant jump rates per data point ${\lambda }_{1}=0.3$ and ${\lambda }_{2}=0.2$ and ${\lambda }_{3}=0.5$, respectively. We generated the synthetic time series $x(t)$ using the discretization of Eq. (24) with a sampling interval $dt=0.001$ in Euler–Maruyama scheme. A random path of $x(t)$, and corresponding increments $y\left(t\right)=x\left(t+\Delta t\right)-x\left(t\right)$ are shown in Fig. 6.

Afterwards, by calculating the statistical moments of $y(t)$ for $n=1, 2, 4, 6, 8, 10, 12$ and substituting in Eqs. (26) and (27), we estimated the drift parameter and jumps characteristics. The obtained results confirm the effectiveness of the presented modeling:

$$\mu \approx 4.9, {\sigma }_{\xi 1}^{2}\approx 0.2001, {\sigma }_{\xi 2}^{2}\approx 0.6010, {\sigma }_{\xi 3}^{2}\approx 9.9928,$$

$${\uplambda }_{1}\approx 0.3002, {\uplambda }_{2}\approx 0.2010, {\uplambda }_{3}\approx 0.4988.$$

## Conclusion

We discussed that when one deals with data sampled at discrete times, one encounters successive discontinuities along the path of the sampled time series. The observation of such sequential discontinuities, in the sample path of empirical time series, gave us the idea to develop a new modeling in which any random variation in the path is attributed to a jump event, even if the sampled time series belongs to the class of diffusive processes. Based on this, we introduced a new dynamical stochastic equation -a jump-jump equation- including a deterministic drift term and a combination of several Poisson jump processes with different distributed sizes. The general form of this equation is as follows:

$$dx\left(t\right)={D}^{\left(1\right)}\left(x\right)dt+\sum_{i=1}^{N}{\xi }_{i}d{J}_{i}\left(t\right)$$

In this modeling we also assumed that the jump events do not occur simultaneously so that the jumps have no overlap. We started with the simplest form of equation including a deterministic drift term and a jump process as the stochastic component, and argued that it can be used to describe the discrete time evolution of a Langevin process. We provided a measure to distinguish the type of underlying process -diffusive or jumpy- from the corresponding time series as well. Afterwards, we increased the variety of modeling by considering more jump processes with different distributed sizes. We also demonstrated that all unknown functions and parameters required for each of the modeling are estimated non-parametrically from the measured data set. It should be noted that depending on the number of data points and variety of the amplitude of fluctuations, the jump-jump equation allows one to keep a greater number of stochastic terms (jump processes) for more accurate modeling. But on the other hand, the more the number of jump processes the need to solve the system of equations with more unknowns, the cost of which should be paid in the form of longer runtime.

## Data Availability

The datasets used and/or analyzed during the current study available from the corresponding author on reasonable request.

## Appendix

### Proof of relations (12)

To prove the relations in Eq. (12), we can find the conditional moments of $dx$ from Eq. (11). The first-order conditional moment of $dx$ is obtained by conditional averaging of Eq. (11) over two independent processes, i.e. Poisson-distributed jumps $dJ$ and jump amplitude $\xi$:

$$\left\langle{dx\left(t\right){|}_{x\left(t\right)=x}}\right\rangle ={D}^{\left(1\right)}\left(x\right)dt+\left\langle{\xi dJ}\right\rangle$$

$$={D}^{\left(1\right)}\left(x\right)dt+\left\langle{\xi }\right\rangle
\left\langle{dJ}\right\rangle$$

With assuming that $\xi$ has a Gaussian distribution with zero mean, i.e. $\left\langle{\xi }\right\rangle =0$, we have:

$$\left\langle{dx\left(t\right){|}_{x\left(t\right)=x}}\right\rangle ={D}^{\left(1\right)}\left(x\right)dt,$$

which is the proof of the first relation in Eq. (12). Similarly, the *n*th-order conditioned moment of $dx$ for $n\ge 2$ leads to:

$$\left\langle{dx{\left(t\right)}^{n}{|}_{x\left(t\right)=x}}\right\rangle = \left\langle{{\left[{D}^{\left(1\right)}\left(x\right)dt+\xi dJ\right]}^{n}}\right\rangle$$

$$=\sum_{k=0}^{n}\left\langle{\left[\left(\genfrac{}{}{0pt}{}{n}{k}\right){\left({D}^{\left(1\right)}\left(x\right)dt\right)}^{n-k}{\left(\xi dJ\right)}^{k}\right]}\right\rangle$$

Up to order of $\mathcal{O}(dt)$ we have:

$$\left\langle{dx{\left(t\right)}^{n}{|}_{x\left(t\right)=x}}\right\rangle =\left\langle{{\xi }^{n}}\right\rangle\lambda \left(x\right)dt,$$

which leads to the last relation in Eq. (12).

### Proof of relations (18)

To prove the relations in Eq. (18), we can find the conditional moments of $dx$ from Eq. (17). The first-order conditional moment of $dx$ is obtained by conditional averaging of Eq. (17) over four independent processes, two Poisson-distributed jumps $d{J}_{1}$ and $d{J}_{2}$ (which we assume do not occur simultaneously) and two jump amplitudes ${\xi }_{1}$ and ${\xi }_{2}$:

$$\left\langle{dx\left(t\right){|}_{x\left(t\right)=x}}\right\rangle ={D}^{\left(1\right)}\left(x\right)dt+\left\langle{{\xi }_{1}d{J}_{1}}\right\rangle+\left\langle{{\xi }_{2}d{J}_{2}}\right\rangle$$

$$={D}^{\left(1\right)}\left(x\right)dt+\left\langle{{\xi }_{1}}\right\rangle
\left\langle{d{J}_{1}}\right\rangle+\left\langle{{\xi }_{2}}\right\rangle
\left\langle{d{J}_{2}}\right\rangle$$

$$={D}^{\left(1\right)}\left(x\right)dt+\left\langle{{\xi }_{1}}\right\rangle{\lambda }_{1}\left(x\right)dt+\left\langle{{\xi }_{2}}\right\rangle{\lambda }_{2}\left(x\right)dt.$$

With assuming that ${\xi }_{1}$ and ${\xi }_{2}$ have zero mean Gaussian distributions i.e. $\left\langle{{\xi }_{1}}\right\rangle =0$ and $\left\langle{{\xi }_{2}}\right\rangle =0$, we have:

$$\left\langle{dx\left(t\right){|}_{x\left(t\right)=x}}\right\rangle ={D}^{\left(1\right)}\left(x\right)dt,$$

which is the proof of the first relation in Eq. (18). The *n*th-order conditioned moment of $dx$ for $n\ge 2$ leads to:

28 $$ \begin{gathered} \left\langle {dx\left( t \right)^{n} |_{x\left( t \right) = x} } \right\rangle { } = \left\langle {\left[ {D^{\left( 1 \right)} \left( x \right)dt + \xi_{1} dJ_{1} + \xi_{2} dJ_{2} } \right]^{n} } \right\rangle \hfill \\ = \mathop \sum \limits_{l,m} \left\langle {\left[ {A_{l,m,k} \left( {D^{\left( 1 \right)} \left( x \right)dt} \right)^{l} \left( {\xi_{1} dJ_{1} } \right)^{m} \left( {\xi_{2} dJ_{2} } \right)^{k} } \right]} \right\rangle , \hfill \\ \end{gathered} $$

where ${A}_{l,m,k}=n!/l!m!k!$, so that $l+m+k=n$. Up to order of $\mathcal{O}\left(dt\right)$ we will have:

$$\left\langle{ dx{\left(t\right)}^{n}{|}_{x\left(t\right)=x}}\right\rangle =\left\langle{{\left({\xi }_{1}d{J}_{1}\right)}^{n}+{\left({\xi }_{2}d{J}_{2}\right)}^{n}}\right\rangle$$

$$=\left\langle{{{\xi }_{1}}^{n}}\right\rangle
\left\langle{{{dJ}_{1}}^{n}}\right\rangle+\left\langle{{{\xi }_{2}}^{n}}\right\rangle
\left\langle{{{dJ}_{2}}^{n}}\right\rangle,$$

using $\left\langle{{\left(d{J}_{i}\right)}^{m}}\right\rangle = {\lambda }_{i}dt$ for the statistical moments of $d{J}_{1}$ and $d{J}_{2}$, we will have:

$${K}^{\left(n\right)}\left(x\right)=\left\langle{{{\xi }_{1}}^{n}}\right\rangle{\uplambda }_{1}\left(x\right)dt+\left\langle{{{\xi }_{2}}^{n}}\right\rangle{\uplambda }_{2}\left(x\right)dt,$$

which leads to the second relation in Eq. (18).

### Proof of relations (25)

To prove the relations in Eq. (25), we can find the statistical moments of $dx$ from Eq. (24). The first-order conditional moment of $dx$ is obtained by conditional averaging of Eq. (24) over six independent processes, three Poisson-distributed jumps $d{J}_{1},$ $d{J}_{2},$ $d{J}_{3}$(which we assume do not occur simultaneously) and three jump amplitudes ${\xi }_{1},$ ${\xi }_{2}$ and ${\xi }_{3}$:

$$\left\langle{dx\left(t\right)}\right\rangle =\mu dt+\left\langle{{\xi }_{1}d{J}_{1}}\right\rangle+\left\langle{{\xi }_{2}d{J}_{2}}\right\rangle+\left\langle{{\xi }_{3}d{J}_{3}}\right\rangle$$

$$=\mu dt+\left\langle{{\xi }_{1}}\right\rangle
\left\langle{d{J}_{1}}\right\rangle+\left\langle{{\xi }_{2}}\right\rangle
\left\langle{d{J}_{2}}\right\rangle+\left\langle{{\xi }_{3}}\right\rangle
\left\langle{d{J}_{3}}\right\rangle$$

With assuming that ${\xi }_{1}$ and ${\xi }_{2}$ and ${\xi }_{3}$ have zero mean Gaussian distributions i.e. $\left\langle {{\xi }_{1}}\right\rangle =0$ and $\left\langle {{\xi }_{2}}\right\rangle =0$ and $\left\langle {{\xi }_{3}}\right\rangle =0$ we have:

$$\left\langle {dx\left(t\right)}\right\rangle =\mu dt,$$

which is the proof of the first relation in Eq. (25). The *n*th-order moments of $dx$ for $n\ge 2$ leads to:

29 $$ \begin{gathered}   \left\langle {dx\left( t \right)^{n} } \right\rangle  = \left\langle {\left[ {\mu dt + \xi _{1} dJ_{1}  + \xi _{2} dJ_{2}  + \xi _{3} dJ_{3} } \right]^{n} } \right\rangle  \hfill \\    = \sum\limits_{{l,m}} {\left\langle {\left[ {A_{{l,m,k,p}} \left( {\mu dt} \right)^{l} \left( {\xi _{1} dJ_{1} } \right)^{m} \left( {\xi _{2} dJ_{2} } \right)^{k} \left( {\xi _{3} dJ_{3} } \right)^{p} } \right]} \right\rangle } , \hfill \\  \end{gathered}  $$

where $${A}_{l,m,k,p}=n!/l!m!k!, $$ so that $$l+m+k+p=n$$. Up to order of $$\mathcal{O}\left(dt\right)$$ we have:

$$\left\langle{ dx{\left(t\right)}^{n}}\right\rangle=\left\langle{{\left({\xi }_{1}d{J}_{1}\right)}^{n}+{\left({\xi }_{2}d{J}_{2}\right)}^{n}+{\left({\xi }_{3}d{J}_{3}\right)}^{n}}\right\rangle$$

$$=\left\langle{{{\xi }_{1}}^{n}}\right\rangle
\left\langle{{{dJ}_{1}}^{n}}\right\rangle+\left\langle{{{\xi }_{2}}^{n}}\right\rangle
\left\langle{{{dJ}_{2}}^{n}}\right\rangle+\left\langle{{{\xi }_{3}}^{n}}\right\rangle
\left\langle{{{dJ}_{3}}^{n}}\right\rangle$$

using $\left\langle{{\left(d{J}_{i}\right)}^{m}}\right\rangle ={\lambda }_{i}dt$ for the statistical moments of three Poisson Jumps, we will have:

$$\left\langle{ dx{\left(t\right)}^{n}}\right\rangle= \left\langle{{{\xi }_{1}}^{n}}\right\rangle{\uplambda }_{1}dt+\left\langle{{{\xi }_{2}}^{n}}\right\rangle{\uplambda }_{2}dt+\left\langle{{{\xi }_{3}}^{n}}\right\rangle{\uplambda }_{3}dt,$$

Which leads to the last relation in Eq. (25).
